# ATR activation is regulated by dimerization of ATR activating proteins

**DOI:** 10.1016/j.jbc.2021.100455

**Published:** 2021-02-24

**Authors:** Vaughn Thada, David Cortez

**Affiliations:** Department of Biochemistry, Vanderbilt University School of Medicine, Nashville Tennessee, USA

**Keywords:** ETAA1, TOPBP1, ATR, DNA damage response, replication stress response, cell cycle checkpoint, phosphatidylinositol 3-kinase related protein kinase, ATR activation domain, RPA, AAD, ATR activation domain, AKT, AKT serine/threonine kinase 1, ATM, ataxia telangiectasia mutated, ATR, ataxia telangiectasia and Rad3-related, ATRIP, ATR interacting protein, BRCT, BRCA1 C terminus, CPT, camptothecin, ETAA1, ETAA1 activator of ATR kinase, FKBP, FK-506 binding protein, HU, hydroxyurea, MBP, maltose binding protein, MRN, MRE11/RAD50/NBS1 complex, RPA, replication protein A, ssDNA, single-stranded DNA, TOPBP1, DNA topoisomerase II binding protein 1

## Abstract

The checkpoint kinase ATR regulates DNA repair, cell cycle progression, and other DNA damage and replication stress responses. ATR signaling is stimulated by an ATR activating protein, and in metazoan cells, there are at least two ATR activators: TOPBP1 and ETAA1. Current evidence indicates TOPBP1 and ETAA1 activate ATR *via* the same biochemical mechanism, but several aspects of this mechanism remain undefined. For example, ATR and its obligate binding partner ATR interacting protein (ATRIP) form a tetrameric complex consisting of two ATR and two ATRIP molecules, but whether TOPBP1 or ETAA1 dimerization is similarly required for ATR function is unclear. Here, we show that fusion of the TOPBP1 and ETAA1 ATR activation domains (AADs) to dimeric tags makes them more potent activators of ATR *in vitro*. Furthermore, induced dimerization of both AADs using chemical dimerization of a modified FKBP tag enhances ATR kinase activation and signaling in cells. ETAA1 forms oligomeric complexes mediated by regions of the protein that are predicted to be intrinsically disordered. Induced dimerization of a “mini-ETAA1” protein that contains the AAD and Replication Protein A (RPA) interaction motifs enhances ATR signaling, rescues cellular hypersensitivity to DNA damaging agents, and suppresses micronuclei formation in ETAA1-deficient cells. Together, our results indicate that TOPBP1 and ETAA1 dimerization is important for optimal ATR signaling and genome stability.

Replication forks frequently encounter obstacles such as DNA damage and transcriptional machinery that impede their progression (1). Stalled replication forks must be stabilized to avoid collapse into double-strand breaks and to facilitate resumption of DNA synthesis. Upon fork stalling, the phosphoinositide-3 kinase-related kinase ataxia telangiectasia and Rad3-related (ATR) activates the replication stress response, which slows cell cycle progression, suppresses new origin firing, and stabilizes stalled replication forks (2).

ATR activation occurs not only in response to replication stress, but also during normal DNA replication and in mitosis (3, 4, 5, 6). ATR signaling in S-phase suppresses CDK1-dependent activation of a mitotic transcriptional network to enforce an S/G2 checkpoint (3), and in mitosis, ATR signaling ensures proper chromosome alignment and segregation and regulates the spindle assembly checkpoint (4, 5).

ATR is recruited to replication forks through its obligate binding partner ATR interacting protein (ATRIP) *via* a direct interaction between ATRIP and replication protein A (RPA) (7, 8). In mitosis, RPA recruits ATR to centromeric R-loops (4). However, ATR localization to RPA-coated ssDNA is not sufficient for ATR activation. Several additional proteins, including an ATR activating protein, must also be recruited and assembled with ATR (2). In metazoan cells, there are at least two ATR activating proteins, TOPBP1 and ETAA1 (9, 10, 11, 12), whereas *S. cerevisiae* contains at least three activators (13, 14, 15, 16). Whether ATR activation occurs *via* TOPBP1 or ETAA1 is dependent on cell cycle phase and the presence of exogenous DNA damage. In response to replication stress caused by replication inhibitors or DNA damaging agents, ATR signaling is predominantly TOPBP1-dependent, whereas ETAA1 is the primary ATR activator during normal DNA replication and in mitosis (3, 5).

TOPBP1 and ETAA1 can activate different ATR signaling pathways in cells, but the mechanism by which these proteins stimulate ATR activity appears to be the same. Both TOPBP1 and ETAA1 possess experimentally defined ATR activation domains (AADs) that are required to bind and activate ATR-ATRIP (9, 10, 11, 12). Although the AADs do not exhibit significant primary sequence similarity, both are predicted to be intrinsically disordered and both possess a critical tryptophan and predicted coiled-coil motif that are essential for ATR binding and activation (17). Additionally, a mutation in ATR that reduces TOPBP1-dependent activation also reduces activation by ETAA1 (10, 18).

Human and yeast ATR-ATRIP complexes form a dimeric butterfly-like structure similar to ataxia telangiectasia mutated (ATM) with two ATR molecules and two ATRIP molecules in a single complex (19, 20, 21, 22, 23), and dimerization of the complex is required for function (24). ATM is activated by the MRE11-RAD50-NBS1 (MRN) complex (25, 26), and like ATM, MRN is a dimer with two MRE11, RAD50, and NBS1 molecules in a single complex (27). In addition, RAD50 dimerization is required for ATM activation (28). Thus, activation of ATM may require dimerization of the ATM activating protein.

TOPBP1 forms oligomeric complexes in cells (29), but how TOPBP1 oligomerization affects ATR activation is not clear. While one study concluded that TOPBP1 dimerization augments ATR signaling (30), another concluded that TOPBP1 oligomerization mediated by AKT phosphorylation reduces ATR signaling (31). In our studies, we observe increased ATR activation when the AADs are fused to a dimeric GST tag but not a monomeric maltose binding protein (MBP) tag. We assessed how AAD dimerization affects ATR activation and found that dimerization of both the TOPBP1 and ETAA1 AADs enhances ATR activation. Additionally, ETAA1 forms oligomeric complexes in cells. Expression of a dimeric, but not monomeric, mini-ETAA1 protein complements the hydroxyurea (HU) and camptothecin (CPT) hypersensitivity of ETAA1-deficient cells and restores genome stability. Our results indicate that ETAA1 and TOPBP1 dimerization is likely important for optimal ATR checkpoint signaling.

## Results

### GST tag enhances ATR activation by AADs

**Figure 1:**
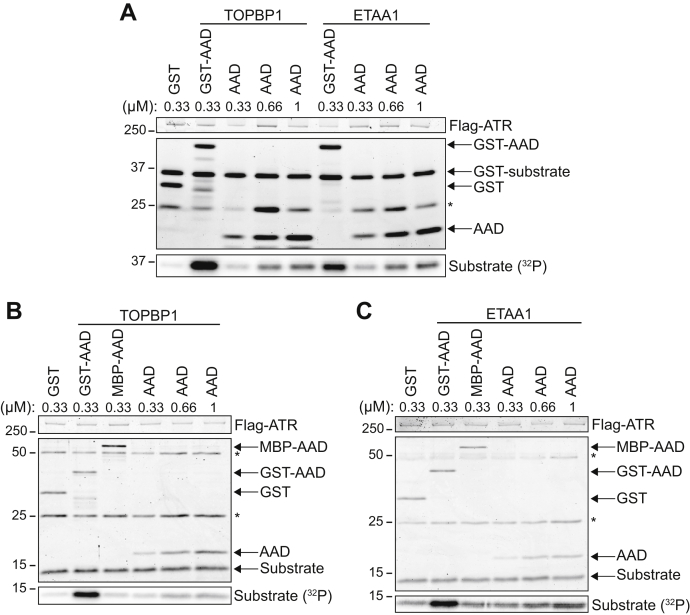
**Fusion of GST to AADs enhances their ability to activate ATR.***A*, the kinase activity of ATR-ATRIP toward a GST-MCM2 substrate was measured in the presence of purified GST, AAD, or GST-AAD proteins. Reaction products were separated by SDS-PAGE and detected by Coomassie blue staining. Substrate phosphorylation was detected by phosphoimaging. *B–C*, kinase activity of ATR-ATRIP toward an untagged MCM2 substrate was measured in the presence of GST-, MBP-, or untagged AADs. *Asterisks* denote antibody heavy and light chains.

We previously identified the minimal TOPBP1 and ETAA1 AADs by determining which TOPBP1 and ETAA1 fragments were capable of activating ATR in an *in vitro* kinase assay (17). All AADs used in that study had a GST tag. Interestingly, we found that cleavage and removal of the GST tag reduces the ability of the AADs to activate ATR. Even at a threefold higher concentration, ATR activation by untagged TOPBP1 or ETAA1 AADs is still less than activation by the corresponding GST-AADs (compare lanes 2 and 5 and 6 and 9) (Fig. 1*A*). The substrate used in this experiment (GST-MCM2 79-138) possesses a GST tag that may form a dimer (32); therefore, we assessed whether increased ATR activation by GST-AADs was due to a GST-mediated interaction between substrate and AAD. To do this, we performed kinase assays with the same substrate lacking the GST tag. Again, compared to GST alone, GST-TOPBP1 AAD strongly stimulates ATR kinase activity towards the untagged substrate, while the untagged TOPBP1 AAD does not (Fig. 1*B*). We also determined how another tag that does not dimerize, MBP, affects ATR activation by the AADs. In contrast to the GST-TOPBP1 AAD, the MBP-TOPBP1 AAD induces minimal, if any, ATR activation toward the untagged substrate (Fig. 1*B*). We observed the same result with GST-tagged, MBP-tagged, and untagged ETAA1 AADs (Fig. 1*C*). Together, these results indicate that a GST tag fused to the TOPBP1 and ETAA1 AADs enhances ATR activation without facilitating an interaction with the kinase substrate.

### AAD dimerization enhances ATR activation

**Figure 2:**
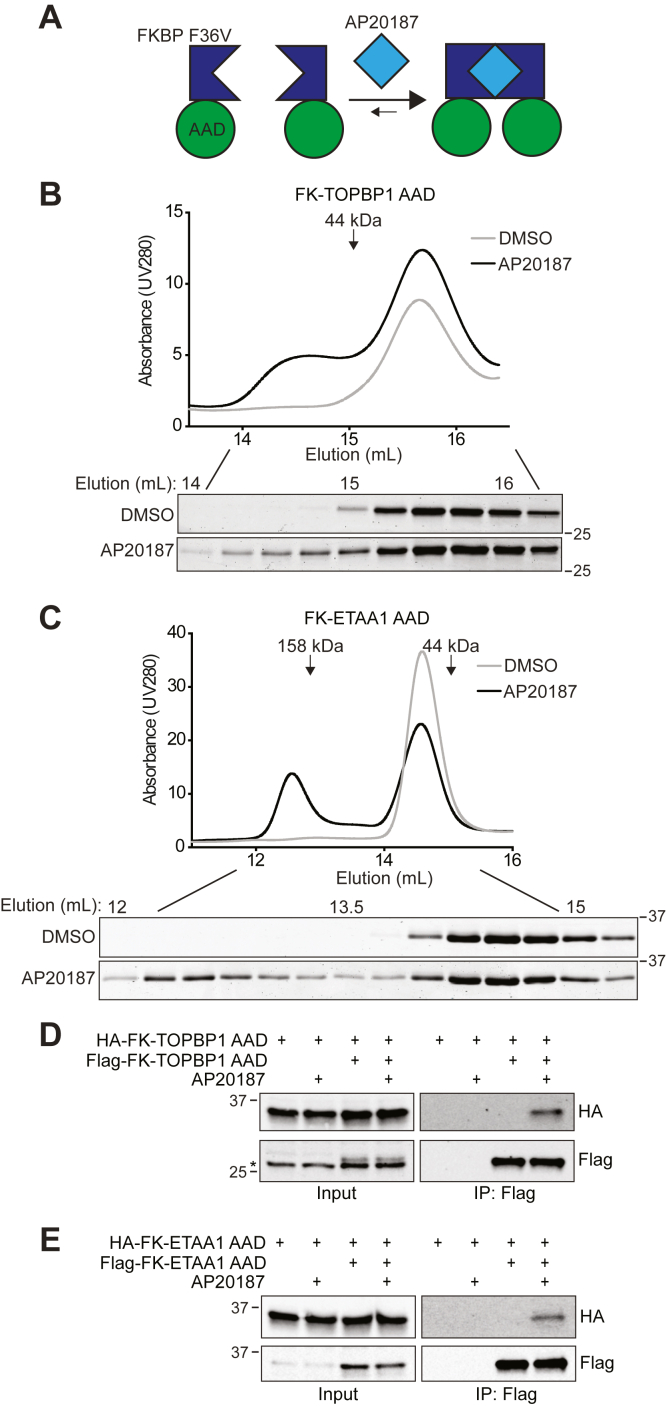
**FKBP F36 V (FK)-AADs dimerize when incubated with AP20187.***A*, schematic depicting the FK inducible dimerization system. *B–C*, purified FK-TOPBP1 AAD (*B*) or FK-ETAA1 AAD (*C*) was preincubated with either DMSO or 5 μM AP20187 for 1 h prior to application to a Superdex 200 Increase 10/300Gl column. Samples from the indicated fractions were analyzed by SDS-PAGE and Coomassie blue staining. *D–E*, the indicated HA- or Flag-tagged proteins were expressed in HEK293T cells. Cells were incubated with DMSO or 100 nM AP20187 for 1 h prior to lysis with buffer containing DMSO or 4 μM AP20187. Immunoprecipitated proteins were separated by SDS-PAGE and detected by immunoblotting. *Asterisk* denotes nonspecific band at similar MW as Flag-FK-TOPBP1 AAD (*D*).

Because GST can form a dimer (32), we hypothesized that GST-mediated AAD dimerization might be the cause of the increased ATR activation. To test this hypothesis directly, we generated AADs with an FKBP F36V tag. Dimerization of proteins fused to FKBP F36V (hereafter referred to as FK) occurs upon incubation with the rapamycin analog AP20187 (Fig. 2*A*) (33). We first determined whether AP20187 induces dimerization of FK-TOPBP1 AAD and/or FK-ETAA1 AAD. In the absence of AP20187, purified FK-TOPBP1 AAD elutes from a size-exclusion column at a retention volume of 15.7 ml, which corresponds to a molecular weight of 29 KDa (Fig. 2*B*). FK-TOPBP1 AAD has a predicted molecular weight of 28.5 KDa, suggesting that in the absence of AP20187, FK-TOPBP1 AAD is a monomer. Upon preincubation with AP20187, a second FK-TOPBP1 AAD elution peak is observed at a retention volume of 14.6 ml (Fig. 2*B*). This elution volume corresponds to a molecular weight of 54 KDa, approximately twice the predicted molecular weight of FK-TOPBP1 AAD. Thus, as expected, AP20187 induces FK-TOPBP1 AAD dimerization.

FK-ETAA1 AAD has a predicted molecular weight of 31.5 KDa, but in the absence of AP20187 elutes from a size-exclusion column at a retention volume (14.6 ml) corresponding to a molecular weight of 54 KDa (Fig. 2*C*). The ETAA1 AAD is predicted to be intrinsically disordered and unlikely to form a globular fold (17), which may explain this result. Upon preincubation with AP20187, FK-ETAA1 AAD elutes at a second peak of 12.6 ml, which corresponds to a predicted molecular weight of 163 KDa (Fig. 2*C*). This result is consistent with AP20187 inducing FK-ETAA1 AAD dimerization or oligomerization.

Like the FK-ETAA1 AAD, we previously found that the untagged ETAA1 and TOPBP1 AADs elute from a size-exclusion column at larger than predicted molecular weights (17). These results could be due to the predicted intrinsic disorder of the AADs or to AAD dimerization. To assess whether the TOPBP1 and ETAA1 AADs dimerize, we performed co-immunoprecipitation experiments. Immunoprecipitation of Flag-FK-TOPBP1 AAD coprecipitates HA-FK-TOPBP1 AAD in the presence, but not the absence, of AP20187 (Fig. 2*D*). The same result is observed when Flag- and HA-FK-ETAA1 AAD are coexpressed in cells (Fig. 2*E*). These results suggest that the AADs alone are not capable of dimerizing and that their elution profiles in size-exclusion chromatography are likely because they do not fold into a globular structure as is predicted from their high degree of disorder in prediction algorithms.

**Figure 3:**
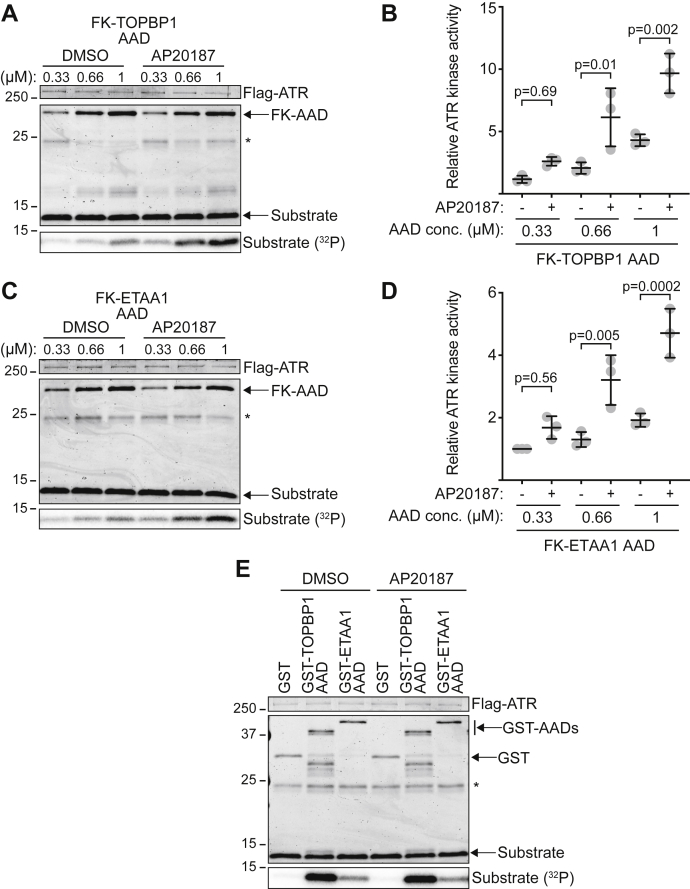
**AAD dimerization enhances ATR activation.***A–D*, kinase activity of ATR-ATRIP toward untagged MCM2 substrate in the presence of FK-TOPBP1 AAD (*A*) or FK-ETAA1 AAD (*C*) was measured in the presence of DMSO or 5 μM AP20187. Reaction products were separated by SDS-PAGE and detected by Coomassie staining. Quantification of substrate phosphorylation in three independent experiments (*B* and *D*) was measured using a phosphoimager. Statistical significance was calculated using a one-way ANOVA and Tukey’s multiple comparisons test (Mean ± SD). *E*, kinase activity of ATR-ATRIP toward untagged MCM2 substrate in the presence of GST or the indicated GST-AADs in the presence of DMSO or 5 μM AP20187. *Asterisks* denote antibody light chain.

Next, we determined how AAD dimerization affects ATR activation by performing kinase assays with the FK-AADs in the absence or presence of AP20187. FK-TOPBP1 AAD activates ATR more efficiently in the presence of AP20187, and this result is observed regardless of AAD concentration (Fig. 3, *A* and *B*). AP20187 also increases ATR activation by the FK-ETAA1 AAD (Fig. 3, *C* and *D*). These results are not due to AP20187 off-target effects since basal ATR activation levels and ATR activation by GST-AADs are not changed in the presence of AP20187 (Fig. 3*E*). Thus, AAD dimerization enhances ATR activation *in vitro*.

### ETAA1 forms oligomeric complexes in cells

**Figure 4:**
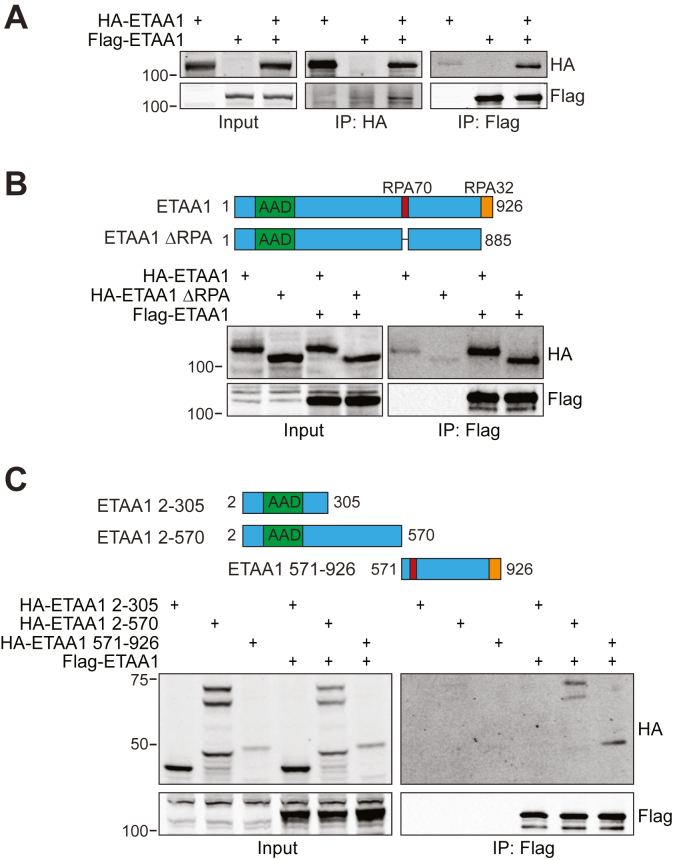
**ETAA1 forms oligomeric complexes in cells.***A*, Flag-ETAA1 and HA-ETAA1 were expressed either alone or in combination in HEK293T cells prior to lysis, immunoprecipitation, and immunoblotting. *B*, HA-ETAA1 or HA-ETAA1 ΔRPA was expressed either alone or in combination with Flag-ETAA1 in HEK293T cells prior to lysis, immunoprecipitation, and immunoblotting. *C*, HA-ETAA1 2–305, 2–570, or 571–926 was expressed either alone or in combination with Flag-ETAA1 in HEK293T cells prior to lysis, immunoprecipitation, and immunoblotting.

Based on our biochemical data, we wondered whether dimerization (or oligomerization) of ATR activating proteins in cells is important for ATR signaling. TOPBP1 oligomerization has been reported to enhance or attenuate ATR signaling (30, 31), but whether ETAA1 oligomerization affects ATR signaling is not known. To determine if ETAA1 forms oligomeric complexes, we expressed Flag-ETAA1 and HA-ETAA1 in cells either alone or in combination and examined potential oligomerization by co-immunoprecipitation. Immunoprecipitation of HA-ETAA1 coprecipitates Flag-ETAA1, and immunoprecipitation of Flag-ETAA1 coprecipitates HA-ETAA1 (Fig. 4*A*). ETAA1 localizes to ssDNA through a direct interaction with RPA (10, 11, 12). Because ETAA1 contains two RPA interaction motifs that interact with two different RPA subunits, we tested whether ETAA1 oligomerization was dependent on the ETAA1-RPA interaction. Immunoprecipitation of Flag-ETAA1 results in similar coprecipitation of HA-ETAA1 and HA-ETAA1 ΔRPA (a mutant lacking both RPA interaction motifs), indicating that ETAA1 oligomerization occurs independently of RPA binding (Fig. 4*B*).

Next, we attempted to identify a discreet oligomerization domain within ETAA1. We expressed HA-ETAA1 fragments 2–305, 2–570, and 571–926 in cells either alone or in combination with Flag-ETAA1. Immunoprecipitation of Flag-ETAA1 coprecipitates both HA-ETAA1 2–570 and 571–926, but not 2–305 (Fig. 4*C*). This data suggests that ETAA1 oligomerization may occur through multiple interaction surfaces.

### Dimeric mini-ETAA1 restores function in ETAA1-deficient cells

**Figure 5:**
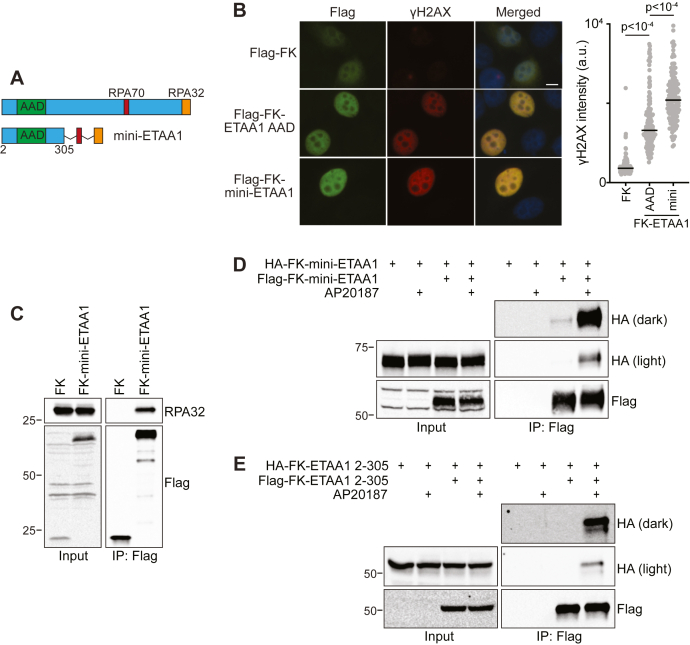
**Creation and characterization of a mini-ETAA1 protein.***A*, schematic comparing ETAA1 to mini-ETAA1. Domains of mini-ETAA1 are separated by a flexible linker (GGGGS)_3_. *B*, HeLa cells expressing either Flag-FK, Flag-FK-ETAA1 AAD, or Flag-FK-mini-ETAA1 in the absence of AP20187 were fixed and stained for Flag and γH2AX. Representative images and γH2AX quantification of at least 100 Flag positive cells per condition are shown. Scale bar is 10 μm. Statistical significance was calculated with a Kruskal–Wallis test and Dunn’s multiple comparisons test. Black bars indicate median. *C*, FK or FK-mini-ETAA1 was expressed in HEK293T cells. Cell were lysed, and immunoprecipitated proteins were detected by immunoblotting. *D*, HA-FK-mini-ETAA1 was expressed either alone or in combination with Flag-FK-mini-ETAA1 in HEK293T cells. Cells were preincubated with DMSO or 100 nM AP20187 for 1 h prior to lysis in buffer containing DMSO or 4 μM AP20187. Immunoprecipitated proteins were detected by immunoblotting. *E*, HA-FK-ETAA1 2–305 was expressed either alone or in combination with Flag-FK-ETAA1 2–305 in HEK293T cells. Cells were preincubated with DMSO or 100 nM AP20187 for 1 h prior to lysis in buffer containing DMSO or 4 μM AP20187. Immunoprecipitated proteins were detected by immunoblotting.

We have been unsuccessful in further narrowing the oligomerization surfaces in part because of the instability of ETAA1 fragments and lack of predicted folded domains. Nonetheless, we were interested in determining if ETAA1 oligomerization affects ATR signaling in cells, so investigated whether we could prevent oligomerization by removing most of the ETAA1 regions predicted to be disordered while retaining known functional motifs. To do this, we created a protein we have termed mini-ETAA1, in which we fused ETAA1 amino acids 2–305 to the RPA70 (residues 600–622) and RPA32 (residues 885–926) interaction motifs (Fig. 5*A*). In addition to the AAD and RPA interaction motifs, mini-ETAA1 also retains CDK-dependent phosphorylation sites that are required for ETAA1-dependent suppression of chromosomal instability (34).

We assessed the functionality of mini-ETAA1 by measuring ATR activation and RPA binding. Overexpression of both full-length ETAA1 and the ETAA1 AAD results in ectopic ATR activation that can be measured by γH2AX induction (10, 17, 34). FK-mini-ETAA1 overexpression causes a large increase in γH2AX induction and an even greater increase in γH2AX than is caused by FK-ETAA1 AAD overexpression (Fig. 5*B*). In addition, immunoprecipitation of FK-mini-ETAA1 coprecipitates RPA32 (Fig. 5*C*). These results indicate FK-mini-ETAA1 is capable of activating ATR and binding RPA.

Next, we examined mini-ETAA1 oligomerization. As expected, immunoprecipitation of Flag-FK-mini-ETAA1 results in coprecipitation of HA-FK-mini-ETAA1 in the presence of AP20187. We also detect a substantially attenuated interaction between Flag- and HA-FK-mini-ETAA1 in the absence of AP20187 (Fig. 5*D*). This small residual interaction is mediated by RPA since immunoprecipitation of Flag-FK-ETAA1 2–305 (which lacks the RPA interaction motifs) only coprecipitates HA-FK-ETAA1 2–305 in the presence, but not absence, of AP20187 (Fig. 5*E*).

**Figure 6:**
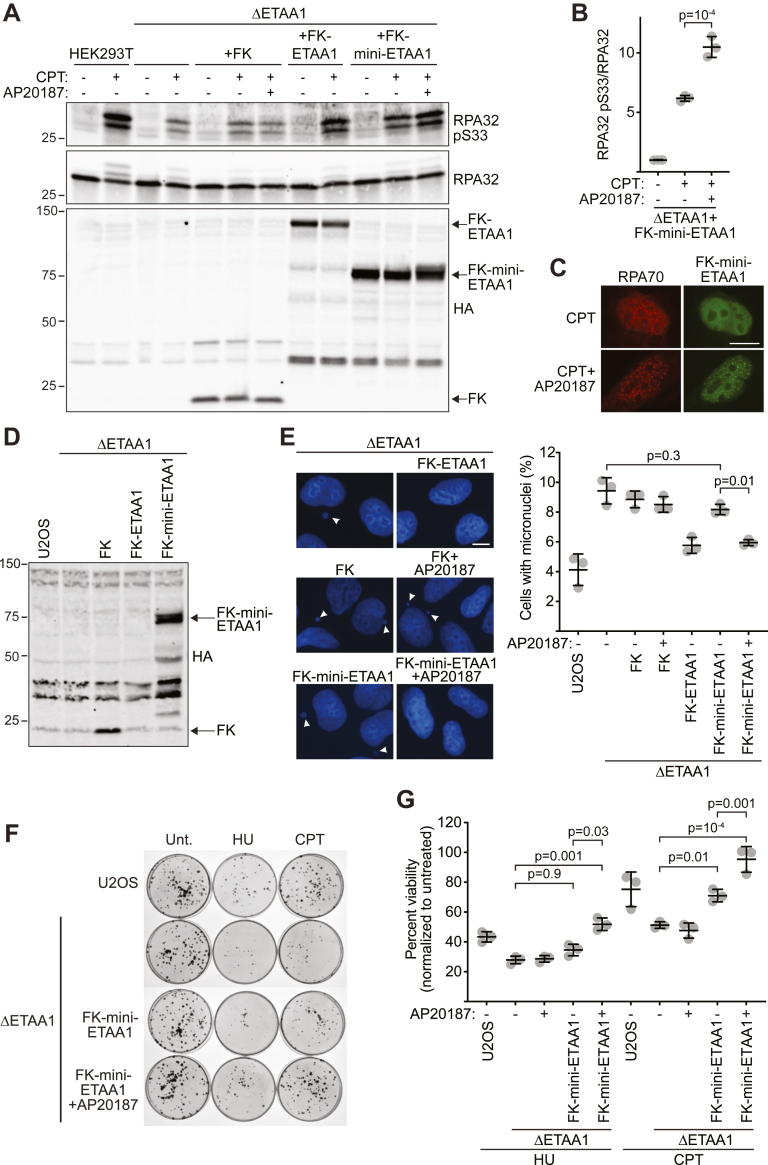
**Induced dimerization of mini-ETAA1 restores function in ETAA1-deficient cells.***A*, HEK293T, HEK293T ΔETAA1, or HEK293T ΔETAA1 cells complemented with the indicated cDNAs were preincubated with 100 nM AP20187 for 2 h before incubation with 300 nM CPT ± 100 nM AP20187 for 6 h as indicated. Cells were lysed and proteins were separated by SDS-PAGE and immunoblotted with the indicated antibodies. *B*, quantification of RPA32 S33 phosphorylation in HEK293T ΔETAA1 cells expressing FK-mini-ETAA1 treated with 300 nM CPT and/or 100 nM AP20187 as indicated. Statistical significance was calculated with a one-way ANOVA and Tukey’s multiple comparisons test (mean ± SD, n = 3). *C*, U2OS cells expressing HA-FK-mini-ETAA1 were treated with 300 nM CPT for 3 h or pretreated with 100 nM AP20187 for 1 h before treatment with 300 nM CPT and 100 nM AP20187 for 3 h. Cells were fixed and stained for RPA70 and HA-FK-mini-ETAA1. Representative images for each condition are shown. Scale bar is 10 μm. *D*, U2OS ΔETAA1 cells expressing HA-FK, HA-FK-ETAA1, or HA-FK-mini-ETAA1 were generated by lentiviral infection. HA-FK-ETAA1 expression is below limit of detection. *E*, micronuclei quantification and representative images in the indicated cell types incubated in the absence or presence of 100 nM AP20187 for 24 h. Scale bar is 10 μm. Statistical significance was calculated with a one-way ANOVA and Tukey’s multiple comparisons test (Mean ± SD, n = 3). *F*, representative images of a clonogenic survival assay of the indicated cell types treated with 1 mM HU ± 100 nM AP20187 or 5 nM CPT ± 100 nM AP20187 for 24 h. *G*, quantification of survival assay shown in *F*. Percent viability for each condition is normalized to the corresponding untreated control. Statistical significance was calculated with a one-way ANOVA and Tukey’s multiple comparisons test (mean ± SD, n = 3 technical triplicate).

We next tested whether monomeric and/or dimeric mini-ETAA1 is sufficient to restore function to ETAA1-deficient cells. First, we assessed ATR-dependent RPA32 phosphorylation in response to CPT, which is reduced in ETAA1-deficient cells (10, 11, 12). As previously reported, ΔETAA1 cells generated by CRISPR-Cas9 gene editing have reduced RPA32 S33 phosphorylation upon CPT treatment compared to wild-type controls or ΔETAA1 cells complemented with FK-full-length ETAA1 (Fig. 6*A*). Expression of the FK tag by itself does not complement the defect whether or not the dimerization ligand AP20187 is added to the culture media (Fig. 6*A*). In contrast, ΔETAA1 cells expressing FK-mini-ETAA1 in the absence of AP20187 exhibit partial restoration of RPA32 phosphorylation upon CPT treatment. Importantly, phosphorylation is further increased in these cells in the presence of AP20187 (Fig. 6, *A* and *B*). This result is likely due to more potent ATR activation by dimeric FK-mini-ETAA1, but also due to increased recruitment of dimeric FK-mini-ETAA1 to damaged replication forks. Indeed, FK-mini-ETAA1 colocalization with RPA in response to CPT is increased upon addition of AP20187 (Fig. 6*C*).

ETAA1-deficient cells also exhibit increased genome instability, including a greater number of cells with micronuclei due to defective ATR signaling (10). To examine micronuclei formation, we first generated U2OS ΔETAA1 stable cell lines expressing either FK, FK-ETAA1, or FK-mini-ETAA1 (Fig. 6*D*). Although we have been unable to detect expression of FK-ETAA1 by immunoblot in these cells, our phenotypic data indicates that it is expressed since it complements ETAA1-deficient phenotypes (Fig. 6, *D* and *E*). As expected, ΔETAA1 cells, or ΔETAA1 cells expressing only the FK protein exhibit increased micronuclei formation irrespective of whether AP20187 is present (Fig. 6*E*). In contrast, ΔETAA1 cells expressing FK-full length ETAA1 have reduced micronuclei formation (Fig. 6*E*). ΔETAA1 cells expressing FK-mini-ETAA1 in the absence of AP20187 have micronuclei levels similar to ΔETAA1 and ΔETAA1+FK cells. However, incubation of ΔETAA1+FK-mini-ETAA1 cells with AP20187 reduces the percentage of cells with micronuclei to the same level as ΔETAA1 cells expressing FK-full length ETAA1 (Fig. 6*E*).

Finally, we examined whether FK-mini-ETAA1 dimerization complements the HU and CPT sensitivity of ΔETAA1 cells. As shown previously, ETAA1 loss decreases viability in a clonogenic survival assay upon HU and CPT treatment (Fig. 6, *F* and *G*). In the absence of AP20187, expression of FK-mini-ETAA1 has minimal impact on HU sensitivity although it does reduce hypersensitivity to CPT. Induction of mini-ETAA1 dimerization by AP20187 further reduces sensitivity to both HU and CPT (Fig. 6, *F* and *G*). These results are not due to other effects of AP20187 as AP20187 does not alter the HU and CPT sensitivity of ΔETAA1 cells (Fig. 6*G*). Altogether, these results indicate that dimerization of mini-ETAA1 restores functionality and largely rescues the genome instability and HU and CPT sensitivities caused by defective ATR signaling in ETAA1-deficient cells.

## Discussion

ATR-ATRIP is a dimer of dimers with two ATR and two ATRIP molecules in a single complex, and this dimerization is required for signaling and checkpoint activity (19, 20, 24). We now find that dimerization of the TOPBP1 and ETAA1 AADs is also important for ATR activation and function. Like TOPBP1, ETAA1 forms oligomeric complexes in cells. An ETAA1 protein that cannot dimerize (mini-ETAA1) is unable to restore function in ETAA1-deficient cells until it is induced to dimerize with a heterologous dimerization domain. These results are consistent with the hypothesis that TOPBP1 and ETAA1 activate ATR as dimers *via* the same biochemical mechanism.

Consistent with this conclusion, ATR activation in *Xenopus* egg extracts was previously shown to be strongly stimulated by a GST-TOPBP1 AAD recombinant protein, but only weakly stimulated by an MBP-TOPBP1 AAD (35). Addition of an FK tag to MBP-TOPBP1 AAD causes increased ATR activation in the presence, but not absence, of AP20187. The TOPBP1 AAD elutes from a size-exclusion column at a larger than predicted molecular weight (17). This difference has been suggested to be because the TOPBP1 AAD itself is a dimer (35). However, our data indicate that the TOPBP1 and ETAA1 AADs only form dimers when fused to heterologous dimerization domains or are embedded within the full-length proteins. Therefore, the aberrant elution profiles of these proteins from size-exclusion columns are likely because they are intrinsically disordered and do not form globular domains.

Previous studies examining how TOPBP1 oligomerization affects ATR activation reported conflicting results. Our findings are consistent with those of Zhou *et al*. (30), who reported that forced TOPBP1 dimerization enhances ATR signaling. In contrast, Liu *et al*. (31) reported that TOPBP1 oligomerization mediated by AKT phosphorylation reduces ATR signaling. These differences could be attributable to differences in the specific oligomeric structures formed in these circumstances. Consistent with our conclusions, a study published during revisions of this article found that TOPBP1 oligomerization occurs *via* liquid–liquid phase separation and that micron-sized TOPBP1 condensates promote ATR activation (36).

Our domain-mapping studies of ETAA1 suggest that it contains at least two separable regions that mediate oligomerization. Similar findings have been reported for TOPBP1 oligomerization. One study found that AKT-dependent AAD phosphorylation mediates TOPBP1 oligomerization *via* the BRCA1 C-terminus 7/8 (BRCT7/8) domains (31), while another study reported that the BRCT1/2 and BRCT4/5 domains mediate TOPBP1 oligomerization (35). Unlike TOPBP1, ETAA1 is mostly devoid of predicted folded domains, and several regions within ETAA1 are predicted to be intrinsically disordered (17). Although disordered regions in other proteins often promote phase separation due to self-assembly (37), a previous report found no evidence of ETAA1 biomolecular condensate formation (36). Thus, elucidating specifically how ETAA1 oligomerization occurs will require additional studies.

FK-mini-ETAA1, when expressed in ΔETAA1 cells, increases RPA32 phosphorylation in response to CPT treatment, reduces micronuclei formation to the level observed in ΔETAA1 cells expressing full-length ETAA1, and restores the HU and CPT sensitivities of ΔETAA1 cells to wild-type levels only upon mini-ETAA1-induced dimerization. Thus, in addition to ATR-ATRIP and RPA binding, ETAA1 dimerization is needed for its function in the DNA damage response. This result also suggests that these ETAA1 biochemical activities may be sufficient to mediate at least some functions of ETAA1. However, the FK-mini-ETAA1 protein is expressed at higher levels than the full-length protein, which could obscure the need for additional domains or motifs in ETAA1. ETAA1 forms complexes with several other DNA damage response proteins (10). Thus, further studies will be needed to determine if there are additional functional motifs within the disordered regions of ETAA1. Nonetheless, the mini-ETAA1 protein may be useful in future structural studies aimed at visualizing the activated form of ATR since mini-ETAA1 lacks most of the disordered regions and is sufficient to induce an active ATR conformation.

In conclusion, dimerization of the ATR activators ETAA1 and TOPBP1 is important for their function in ATR activation. Regulation of TOPBP1 and ETAA1 oligomerization, possibly mediated by posttranslation modifications and/or phase separation, may facilitate precise spatiotemporal control of ATR signaling.

## Experimental procedures

### Cell culture

U2OS, HEK293T, and HeLa cells were cultured in DMEM +7.5% FBS at 37 °C and 5% CO_2_. Transfections were performed with polyethyleneimine. HEK293T ΔETAA1 and U2OS ΔETAA1 cells were described previously (10), and complementation of U2OS ΔETAA1 cells with FKBP F36V expression constructs was achieved by lentiviral infection followed by selection for the linked puromycin resistance cassette.

### GST protein purification

GST-tagged proteins were purified from ArcticExpress *Escherichia coli* (Agilent Technologies). Bacterial pellets were resuspended in NET buffer (25 mM Tris pH 8, 50 mM NaCl, 0.1 mM EDTA, 5% glycerol, 5 μg/ml aprotinin, 5 μg/ml leupeptin, 1 mM DTT) and sonicated. Triton X-100 was added to a final concentration of 1% and lysates were incubated on ice for 30 min. Following centrifugation, the cleared supernatant was incubated with glutathione-sepharose beads (GE Healthcare, 17-0756-01) for 2.5 h at 4 °C. Beads were washed three times with NET buffer containing 1% Triton X-100, and bound proteins were recovered with elution buffer (75 mM Tris pH 8, 15 mM GSH, 5 μg/ml leupeptin, 1 mM DTT). In experiments where the GST tag was removed, beads were washed twice with cleavage buffer (50 mM Tris pH 7, 150 mM NaCl, 1 mM EDTA, 1 mM DTT) and then incubated with cleavage buffer containing Prescission protease (GE Healthcare, 270843) overnight at 4 °C. Proteins were then dialyzed (20 mM HEPES-KOH pH 7.5, 50 mM NaCl, 1 mM DTT) for 2 h and then again overnight at 4 °C.

### His protein purification

His-MBP-AADs and His-FKBP F36V-AADs were purified from ArcticExpress *E. coli*. Cells were resuspended in Native Purification Buffer (50 mM NaH_2_PO_4_, 500 mM NaCl, 10 mM imidazole, 5 μg/ml aprotinin, 5 μg/ml leupeptin, 4 mm DTT) containing lysozyme and incubated on ice for 30 min. Cells were sonicated and lysates were cleared by centrifugation. Cleared lysates were incubated with Ni-NTA agarose beads (Invitrogen, R901–01) for 3 h at 4 °C. Beads were washed four times with native wash buffer (50 mM NaH_2_PO_4_, 500 mM NaCl, 20 mM imidazole, 5 μg/ml aprotinin, 5 μg/ml leupeptin, 4 mm DTT). Bound proteins were recovered by incubation with native elution buffer (50 mM NaH_2_PO_4_, 500 mM NaCl, 250 mM imidazole, 5 μg/ml aprotinin, 5 μg/ml leupeptin, 4 mm DTT) and dialyzed (20 mM HEPES-KOH pH 7.5, 50 mM NaCl, 1 mM DTT) for 2 h and then again overnight at 4 °C.

### Kinase assays

Flag-ATR–HA-ATRIP complexes were purified from HEK293T cell nuclear extracts with monoclonal anti-HA agarose beads (Sigma-Aldrich, A2095). Beads were washed three times with TGN buffer (50 mM Tris pH 7.5, 150 mM NaCl, 10% glycerol, 1% Tween20, 5 μg/ml aprotinin, 5 μg/ml leupeptin, 1 mM sodium orthovanadate, 10 mM β-glycerol phosphate, 1 mM NaF, 0.5 mM DTT) and once with TGN buffer containing 500 mM LiCl. Beads were then washed twice with kinase buffer (10 mM HEPES pH 7.5, 50 mM NaCl, 10 mM MgCl_2_, 10 mM MnCl_2_, 50 mM β-glycerol phosphate, 1 mM DTT). ATR-ATRIP bound beads were incubated with GST, a GST-AAD, MBP-AAD, FKBP F36V-AAD, or untagged AAD, a GST- or untagged substrate, and [γ-^32^P]ATP for 20 min at 30 °C. Reactions were stopped by addition of 2× SDS sample buffer prior to protein separation by SDS-PAGE and detection by Coomassie staining. Substrate phosphorylation was detected by phosphoimaging. In experiments performed with FKBP F36V-AADs, the FKBP F36V-AADs were incubated with DMSO or 5 μM AP20187 (Clontech, 635058) for 1 h at room temperature prior to beginning kinase reactions. Kinase reactions also contained either DMSO or 5 μM AP20187.

### Size-exclusion chromatography

Purified FKBP F36V-AADs were incubated with DMSO or 5 μM AP20187 for 1 h at room temperature prior to being loaded onto a Superdex 200 Increase 10/300Gl Column (GE Healthcare) equilibrated with buffer (20 mM HEPES pH 7.5, 50 mM NaCl, 10 mM DTT). Proteins were eluted at a rate of 0.25 ml/minute and 0.25 ml fractions were collected. Equal amounts of fractions corresponding to peaks were separated by SDS-PAGE, and proteins were detected by Coomassie staining.

### Co-immunoprecipitation

HEK293T cells were lysed in NP-40 lysis buffer (50 mM Tris pH 7.5, 150 mM NaCl, 10% glycerol, 0.5% NP-40, 5 μg/ml aprotinin, 5 μg/ml leupeptin, 1 mM sodium orthovanadate, 10 mM β-glycerol phosphate, 1 mM NaF, 1 mM DTT) for 30 min on ice and cleared by centrifugation. Supernatants were incubated with EZ View Red Anti-Flag M2 affinity gel (Sigma-Aldrich, F2426) or monoclonal anti-HA agarose beads for 1–2 h at 4 °C. Beads were then washed three times with NP-40 lysis buffer and once with Flag elution buffer (10 mM HEPES-KOH pH 7.9, 300 mM KCl, 1.5 mM MgCl_2_, 0.05% NP-40, 0.5 mM DTT). Proteins bound to EZ View Red Anti-Flag affinity gel were eluted with Flag elution buffer containing 0.3 mg/ml 3× Flag peptide (Sigma-Aldrich, F4799). Immunoprecipitated proteins were separated by SDS-PAGE and detected by immunoblotting. Antibodies used were Flag (1:1000, Sigma-Aldrich, F7425), HA (1:2000, Roche, 11867423001), HA (1:1000, Biolegend, 901501), and RPA32 (1:1000, Abcam, 2175). In some experiments, 0.5% NP-40 was substituted with 0.75% CHAPS. For experiments assessing inducible dimerization, cells were incubated with 100 nM AP20187 for 1 h prior to harvesting, and lysis and elution buffers contained 4 μM AP20187.

### Cell lysis and immunoblotting

Cells were treated as indicated in the appropriate figure legends and then lysed in CHAPS lysis buffer (50 mM Tris pH 7.5, 150 mM NaCl, 10% glycerol, 0.75% CHAPS, 5 μg/ml aprotinin, 5 μg/ml leupeptin, 1 mM sodium orthovanadate, 10 mM β-glycerol phosphate, 1 mM NaF, 1 mM DTT, 1 mM MgCl_2_, 1 μl/ml Pierce universal nuclease for cell lysis) for 20 min on ice and cleared by centrifugation. Equal amounts of lysates were separated by SDS-PAGE and proteins were detected by immunoblotting. Antibodies used were HA, RPA32, and RPA32 pS33 (1:1000, Bethyl, A300–246A). Quantification of immunoblots was performed with Image Lab 3.0 software.

### Immunofluorescence

Cells were fixed with 3% paraformaldehyde/2% sucrose in PBS, washed with PBS, permeabilized with 0.5% Triton X-100 in PBS, washed with PBS, and blocked with 5% BSA in PBS prior to incubation with antibodies and staining with DAPI. Antibodies used were Flag M2 (1:200, Sigma-Aldrich, F3165), γH2AX (1:200, Cell Signaling, 2577), RPA70 (1:200, Cell Signaling, 2267), and HA (1:200, Biolegend, 901501). Images were obtained using a Nikon microscope and nuclear γH2AX intensity was quantitated using Elements software.

### Micronuclei assays

Cells were untreated or treated with 100 nM AP20187 for 24 h prior to fixation with 3% paraformaldehyde/2% sucrose in PBS. Cells were washed with PBS, permeabilized with 0.5% Triton X-100 in PBS, washed with PBS, and stained with DAPI. Cells were imaged using a Nikon microscope and micronuclei were quantitated manually. All samples were blinded to the experimenter.

### Clonogenic survival assays

U2OS, U2OS ΔETAA1, and U2OS ΔETAA1+FK-mini-ETAA1 cells were plated, left untreated, treated with 1 mM HU ± 100 nM AP20187, or treated with 5 nM CPT ± 100 nM AP20187 for 24 h. Following drug removal, cells were allowed to grow for 10 days before staining with methylene blue solution (48% methanol, 2% methylene blue, 50% water). The number of colonies on each plate was quantitated manually and for each condition, percent viability was normalized to viability of the corresponding untreated control.

### Statistical analysis

Statistical analyses were performed using Prism. Specific statistical tests used are indicated in the appropriate figure legends.

## Data availability

All data are contained within the article.

## Conflict of interest

The authors declare that they have no conflicts of interest with the contents of this article.
